# Ligand Bound β1 Integrins Inhibit Procaspase-8 for Mediating Cell Adhesion-Mediated Drug and Radiation Resistance in Human Leukemia Cells

**DOI:** 10.1371/journal.pone.0000269

**Published:** 2007-03-07

**Authors:** Doris Estrugo, Alexander Fischer, Franziska Hess, Harry Scherthan, Claus Belka, Nils Cordes

**Affiliations:** 1 OncoRay–Center for Radiation Research in Oncology, Medical Faculty Carl Gustav Carus, University of Technology Dresden, Dresden, Germany; 2 Bundeswehr Institute of Radiobiology, Munich, Germany; 3 Department of Radiation Oncology, University Tübingen, Tübingen, Germany; Sanofi-Aventis, United States of America

## Abstract

**Background:**

Chemo- and radiotherapeutic responses of leukemia cells are modified by integrin-mediated adhesion to extracellular matrix. To further characterize the molecular mechanisms by which β1 integrins confer radiation and chemoresistance, HL60 human acute promyelocytic leukemia cells stably transfected with β1 integrin and A3 Jurkat T-lymphoma cells deficient for Fas-associated death domain protein or procaspase-8 were examined.

**Methodology/Principal Findings:**

Upon exposure to X-rays, Ara-C or FasL, suspension and adhesion (fibronectin (FN), laminin, collagen-1; 5–100 µg/cm^2^ coating concentration) cultures were processed for measurement of apoptosis, mitochondrial transmembrane potential (MTP), caspase activation, and protein analysis. Overexpression of β1 integrins enhanced the cellular sensitivity to X-rays and Ara-C, which was counteracted by increasing concentrations of matrix proteins in association with reduced caspase-3 and -8 activation and MTP breakdown. Usage of stimulatory or inhibitory anti β1 integrin antibodies, pharmacological caspase or phosphatidylinositol-3 kinase (PI3K) inhibitors, coprecipitation experiments and siRNA-mediated β1 integrin silencing provided further data showing an interaction between FN-ligated β1 integrin and PI3K/Akt for inhibiting procaspase-8 cleavage.

**Conclusions/Significance:**

The presented data suggest that the ligand status of β1 integrins is critical for their antiapoptotic effect in leukemia cells treated with Ara-C, FasL or ionizing radiation. The antiapoptotic actions involve formation of a β1 integrin/Akt complex, which signals to prevent procaspase-8-mediated induction of apoptosis in a PI3K-dependent manner. Antagonizing agents targeting β1 integrin and PI3K/Akt signaling in conjunction with conventional therapies might effectively reduce radiation- and drug-resistant tumor populations and treatment failure in hematological malignancies.

## Introduction

Integrin-mediated interactions of cells with extracellular matrix (ECM) are well known to confer resistance to clinically administered chemotherapeutic drugs or ionizing radiation [1]–[8]. These interactions mediate a substantial survival advantage particularly in isolated tumor cell niches. These residual tumor cell islands are likely to represent the starting base for the propagation of highly chemo and radiation resistant clonal cells in hematological neoplasias as well as solid tumors [9].

Twenty-four different α/β heterodimeric transmembrane integrin receptors are formed by 18 α and 8 β integrin subunits, which control survival, apoptosis, proliferation and differentiation among other functions in cooperation with receptor-mediated signaling from soluble growth factors or cytokines [10]. As integrins lack intrinsic kinase activity, different cytoplasmic protein kinases recruited to cytoplasmic integrin domains such as integrin-linked kinase (ILK), focal adhesion kinase (FAK) and phosphatidylinositol-3 kinase (PI3K)/Akt have been reported to transmit signals in normal epithelial cells directly via the PI3K/Akt cascade to prevent anoikis (apoptosis upon detachment from ECM) [11]–[15]. FAK- and NFκB-dependently, integrin-mediated adhesion regulates the expression of several members of the antiapoptotic Bcl-2 protein family [16]–[18]. By downregulating Bim and Bax and upregulating Bcl-2-like proteins, integrin-mediated cell adhesion confers resistance in leukemia cells to genotoxic agents such as Ara-C, bleomycin, fludarabine or ionizing radiation [3], [19]–[21]. The first cue that procaspase-8 might play a critical role in integrin-mediated survival came from studies focusing on integrin-mediated death, which is induced by unligated integrins [22]. It was reported that procaspase-8 binds to the cytoplasmic tail of β integrins. Inhibition of procaspase-8 cleavage via enhanced binding of c-Fas-associated death domain-like interleukin-1-converting enzyme-like inhibitory protein-long (c-FLIP_L_) to Fas-associated death domain protein (FADD) also essentially contributes to adhesion-mediated survival in endothelial cells [23] or mediates drug resistance in myeloma cell lines [24].

Concerning the role of β1 integrins in adherent growing tumor and normal cells, we uncovered a signaling pathway different from the apoptosis cascades. A PI3K-dependent signaling cascade from β1 integrin to the p130Cas/Paxillin/c-Jun N2-terminal kinase complex has demonstrated to confer an advantage of clonogenic cell survival in genotoxically stressed normal fibroblasts and cells from solid tumors [25], [26]. With regard to drug- or radiation-induced apoptosis in leukemia cells such as HL60, ILK promotes apoptosis upon irradiation via caspase-8 or -9 in an adhesion-dependent manner [21]. In HL60 cells, Kasahara et al. [27] have found that FAK, as another critical mediator of integrin signals, functions in a prosurvival manner upon exposure to X-rays. Despite of this interesting discrepancy between ILK and FAK for cell survival after genotoxic stress, we strongly focused on β1 integrin and the intrinsic and extrinsic apoptotic pathways in this study.

In addition to anoikis, there is a large number of different apoptosis-inducing stimuli such as ionizing radiation or cytotoxic drugs. Radiation-induced genotoxic injury mainly triggers the mitochondrial cascade involving release of cytochrome c, dATP, Apaf-1 and procaspase-9 upon Bax translocation to the mitochondrial membrane that, subsequently, results in breakdown of the mitochondrial transmembrane potential (ΔΨm) and autoproteolytic cleavage of caspases [28]–[30]. The extrinsic apoptotic pathway is activated, for example, by binding of the trimeric transmembrane tumor necrosis factor (TNF) family member protein FasL to Fas receptor (FasR) [31]. Subsequently, receptor oligomerization initiates the recruitment of FADD to FasR and procaspase-8 for creating a functional death-inducing signaling complex (DISC) that activates procaspase-3 [32]. Recent studies using FADD [33], procaspase-8 [34] or procaspase-9 [35] knockout mice clearly showed that the FADD/procaspase-8 signaling cascade is central and probably non-redundant in FasR-mediated cell death. While proapoptotic FasL/FasR signaling is promoted by PI3K/Akt in mouse epidermal Cl41 cells [36], PI3K/Akt acts in an antiapoptotic manner in human hepatocytes, [37]. At the level of procaspase-8 or -9, the PI3K/Akt cascade inhibits both the extrinsic and intrinsic apoptotic pathways [38].

In view of the role that cell adhesion-mediated drug and radiation resistance may play in treatment failure and reduced tumor control, it becomes necessary to uncover the integrin-specific molecular mechanisms responsible for evading apoptosis. We therefore examined FasL-, radiation- and Ara-C-induced apoptosis in suspension or adhesion cultures of HL60 acute promyelocytic leukemia and Jurkat T-lymphoma cells with emphasis on integrin β1, procaspase-8 and Akt. Overexpression of the integrin β1 subunit in HL60 cells was used as a model to identify critical signaling pathways participating in the antiapoptotic action of this integrin upon cell adhesion to β1 integrin ligands such as fibronectin and collagen-1. Evidence is provided showing that a) elevated cell surface expression levels of β1 integrins inevitably require elevated amounts of ligands to act in an antiapoptotic manner, and, b) a complex formation of β1 integrin with Akt prevents procaspase-8-mediated apoptosis PI3K-dependently. These data describe a novel mechanism how the integrin β1 facilitates resistance to apoptosis induced by FasL, Ara-C and ionizing radiation, which have different modes of action.

## Results

### Matrix proteins modulate apoptosis and long-term survival after radiation and Ara-C

**Figure 1:**
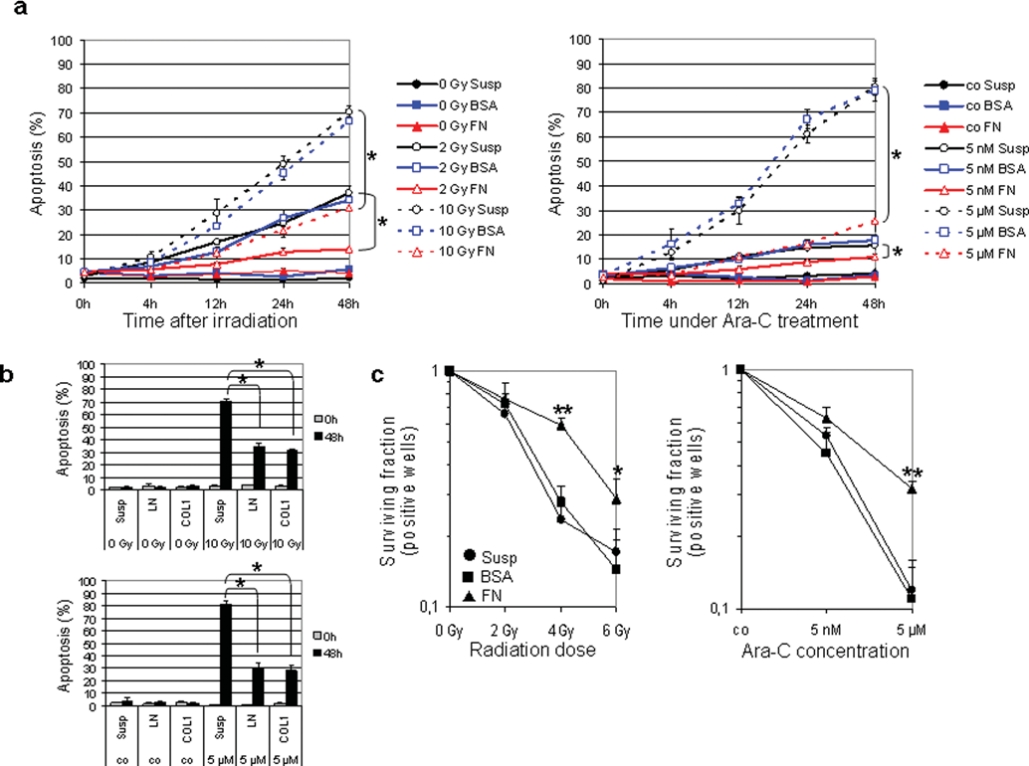
Adhesion to matrix proteins significantly decreases induction of apoptosis in human HL60 acute promyelocytic leukemia cells after irradiation or Ara-C. (a) At 48 h after treatment in suspension (Susp) or on BSA or FN (5 µg/cm^2^), cells were harvested and the number of apoptotic cells was determined by DAPI staining and counting of cells with typically apoptotic nuclear morphology. (b) Apoptosis was also determined in irradiated (10 Gy) or Ara-C (5 µM) treated HL60 cells grown on 5 µg/cm^2^ laminin (LN) or collagen-1 (COL1) after 48 h. (c) Limiting dilution analysis was performed to measure long-time survival. The number of positive wells (i.e. viable and proliferating cells) was used for calculation of survival rates after ionizing radiation (2, 4 or 6 Gy) or a 48-h Ara-C treatment (5 nM or 5 µM) relative to untreated controls (0 Gy or co). Results represent mean±s.d. of three independent experiments. Statistics were calculated by comparing adhesion cultures to matrix proteins versus BSA and/or suspension cultures. *P<0.01.

To assess the impact of fibronectin (FN), laminin (LN) or collagen-1 (COL1) adhesion on short- and long-term survival, HL60 cells were grown in suspension or on BSA, FN, LN or COL1 prior to irradiation or Ara-C. Upon treatment, HL60 adhesion cultures on FN, LN or COL1 showed significant (*P*<0.01) reduction in apoptosis relative to BSA or suspension (Figure 1a and b). Similarly, long-term survival was significantly (*P*<0.01) improved after 4 Gy or 6 Gy or 5 µM Ara-C (Figure 1c). These data clearly indicate that cell-matrix interactions improve survival of HL60 leukemia cells treated with cytotoxic agents that have different modes of action.

### FN concentration determines antiapoptotic effects of β1 integrin

**Figure 2:**
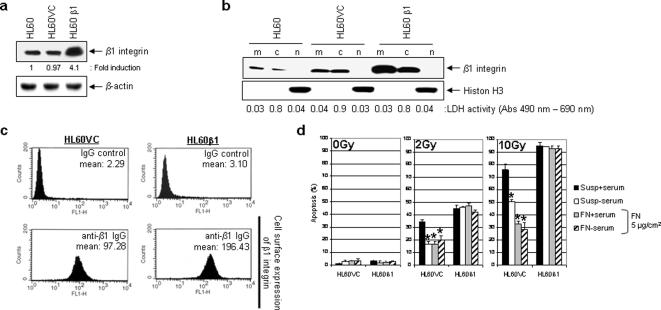
Overexpression of β1 integrins mediates antiapoptotic effects in irradiated HL60 leukemia cells. (a) HL60 cells were stably transfected with full-length β1 integrin (HL60β1) or empty vector (HL60VC) as indicated by Western blot analysis. β-actin served as loading control. (b) Fractionation of membrane (m), cytoplasmic (c) and nuclear (n) proteins was carried out to analyze the distribution of β1 integrins in the transfected and control cells. Cells were lysed in different buffers and centrifuged according to Materials and Methods. Each protein fraction separated by Western blotting contains the protein amount from 2×10^5^ cells. Histon H3 was detected for the nuclear fraction and a lactate dehydrogenase (LDH) assay was performed on the cytoplasmic fraction. Numbers shown indicate the absorbance of the cytoplasmic protein fraction monitored at 490 nm and 690 nm using a spectral-photometer. (c) Level of cell surface expression of transfected β1 integrin in HL60 cells. β1 integrins were stained with FITC-conjugated anti-β1-integrin antibodies and analyzed by flow cytometry. As control, a FITC-conjugated, isotype-matched non-specific IgG (IgG control) was used. (d) Induction of apoptosis in serum grown or serum-free HL60β1 and HL60VC suspension (susp) or FN (5 µg/cm^2^) cell cultures was examined at 48 h after 2 or 10 Gy (mean±s.d.; n = 3). Student́s *t* test compared FN+serum, FN-serum or susp-serum versus susp+serum cultures. *P<0.01

We next assessed the role of β1 integrin by stable overexpression in HL60 cells leading to an elevation in total as well as in cell surface expression of this integrin subunit as determined by Western blotting on total protein extracts (Figure 2a) and on cytoplasmic, membrane and nuclear protein fractions (Figure 2b) and by FACS analysis (Figure 2c). We hypothesized that an overexpression of this integrin reduces the rate of apoptosis upon cytotoxic stimuli. Unexpectedly, overexpression of β1 integrin (HL60β1) pronouncedly induced apoptosis after irradiation in suspension and on 5 µg/cm^2^ FN relative to HL60 vector controls (HL60VC) (Figure 2d). In suspension, induction of apoptosis in irradiated cells was serum dependent (Figure 2d); a finding not further followed on in this study.

**Figure 3:**
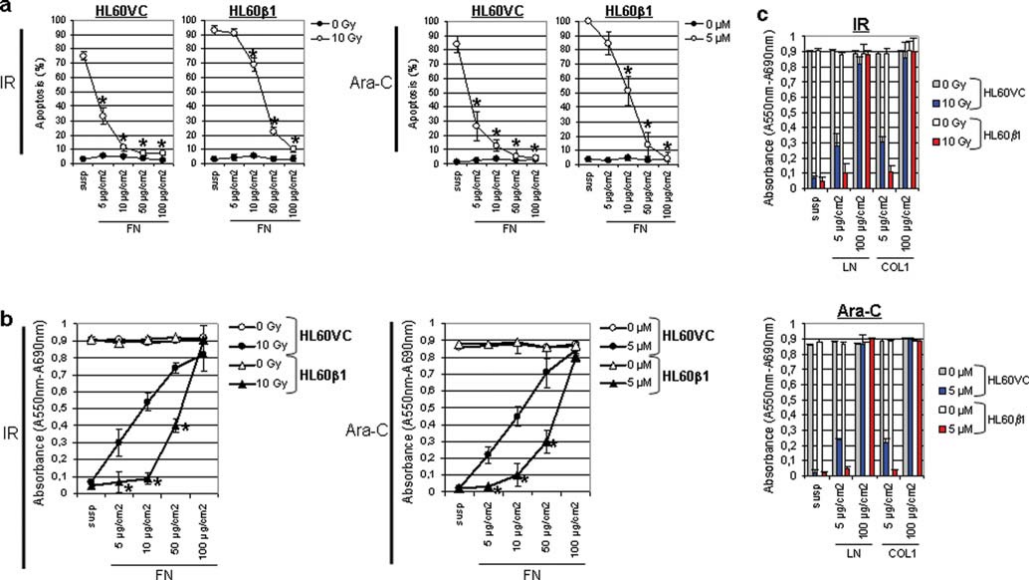
Integrin-mediated cellular resistance to X-rays and Ara-C depends on matrix protein concentrations. (a) After growth in suspension or FN adhesion in serum-free medium for 1 h, cells were exposed to 10 Gy X-rays or 5 µM Ara-C and apoptosis was measured 48 h thereafter (mean±s.d.; n = 3). Statistical analysis compared FN versus suspension cultures. *P<0.01. (b) and (c) Cell viability was determined by MTT assay (see Materials and Methods). Cells (3×10^4^) were seeded onto FN, LN or COL1 in triplicate and grown under similar conditions as described for (a). Experiments were repeated three times and results show mean±s.d.. Statistical analysis compared HL60β1 versus HL60VC cells. *P<0.01. (d) Adhesion of HL60 transfectants to FN was evaluated in the presence or absence of stimulatory (TS2/16; 1 µg/ml) or inhibitory (13; 1 µg/ml) anti-β1 integrin mAbs or peptides (GRGDS; 500 µg/ml) under serum-free conditions (controls: non-specific anti rat IgG1 or GRADSP employed at equivalent concentrations). Columns represent mean±s.d. of the absorbance at 630 nm representing cell adhesion (n = 3). *P*-values were calculated by comparison of mAbs or peptide versus controls. *P<0.01. (e) Radiation-induced apoptosis was determined in cells grown in suspension or on FN in the presence of TS2/16 or mAb13 (1 µg/ml; anti rat IgG1 as control) or GRGDS peptide (500 µg/ml; GRADSP as control) under serum-free conditions. Results represent mean±s.d. (n = 3) and statistics compared mAb or peptide versus controls. *P<0.01.

To clarify the adverse effect of β1 integrin-related enhancement of radiation-induced apoptosis, HL60β1 transfectants were cultured on increasing FN concentrations under serum-free conditions (Figure 3a). While HL60VC cells revealed significantly (*P*<0.01) less apoptosis starting at 5 µg/cm^2^ FN, radiation- and Ara-C-induced HL60β1 apoptosis declined not before 10 µg/cm^2^ FN (Figure 3a). Performing MTT assays in cells cultured on increasing concentrations of FN, LN or COL1 gave results consistent with the apoptosis data sets (Figure 3b and c). These data suggest that only ligand bound β1 integrin functions in an antiapoptotic manner. This issue was further addressed by applying stimulatory or inhibitory anti-β1 integrin mAbs and peptides. HL60VC and HL60β1 cells adhered to FN but not the widely used control substratum BSA (Figure 3d). Incubation of cells with activating mAb TS2/16 promoted adhesion to FN while inhibitory mAb13 significantly (*P*<0.01) impaired adhesion relative to non-specific IgG controls. Adhesion-blocking GRGDS peptides effectively prevented adhesion of both cell lines to FN in contrast to GRADSP (Figure 3d). Under suspension, β1 integrin-activating TS2/16 caused a significant (*P*<0.01) decrease in apoptosis after 10 Gy as compared to IgG (Figure 3e). On 5 or 100 µg/cm^2^ FN, this TS2/16-related antiapoptotic effect further increased while mAb13 strongly promoted apoptosis in irradiated cells.

### Caspase activation and ΔΨm are influenced by FN-β1 integrin interactions

**Figure 4:**
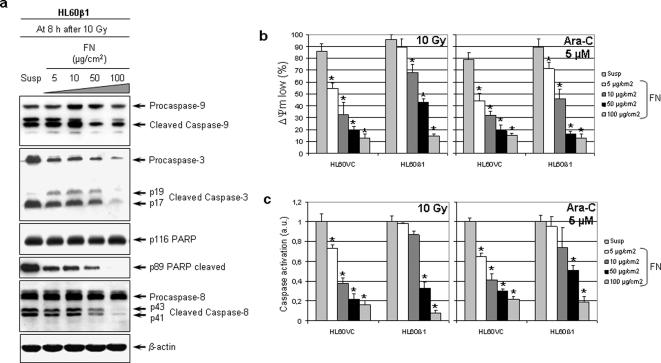
Adhesion to FN reduces radiation-induced cleavage of procaspase-9, -3, -8 and PARP in a concentration dependent manner. (a) Following a 1-h growth on either increasing FN concentrations or in suspension, HL60β1 cells were irradiated with 10 Gy. Cells were harvested 8 h later and total proteins were extracted. After SDS-PAGE and Western blotting, selected proteins were detected using specific antibodies. β-actin served as loading control. (b) FN adhesion maintains the ΔΨm. TMRE staining of 10-Gy irradiated or Ara-C-treated (5 µM) HL60VC and HL60β1 cells was analyzed by FACS to determine the amount of ΔΨm low (mean±s.d.) representing the apoptosis-related breakdown of this potential relative to non-irradiated or non-Ara-C-treated controls ( = 0%). (c) Activation of caspases was determined by FACS analysis in FITC-VAD-fmk-stained cells under identical conditions. Results (mean±s.d. of three independent experiments) are plotted as arbitrary units (a.u.) showing the fold increase after normalization to suspension conditions. Statistics were calculated by comparison of increasing FN concentrations versus suspension. *P<0.01.

To evaluate β1 integrin-dependent regulation of caspase and PARP cleavage after radiation or Ara-C, cells were analyzed on increasing FN concentrations or in suspension. At 8 h after treatment, increasing FN concentrations incrementally reduced cleavage of procaspase-9, -3 and -8 and PARP in adherent, 10-Gy irradiated HL60β1 cells relative to suspension (Figure 4a). p116 PARP and procaspase-8 and -9 expression remained unaffected while procaspase-3 expression slightly declined with increasing FN concentrations. In parallel, ΔΨm (Figure 4b) and caspases activation (Figure 4c) were pronouncedly reduced by increasing FN concentrations in irradiated or Ara-C-treated cells. Again, HL60β1 cells reacted not until higher FN concentrations, i.e. 50 and 100 µg/cm^2^, particularly under Ara-C. In the following, we focused on interactions of β1 integrin with procaspase-8.

### β1 integrin interacts with procaspase-8 and Akt in an adhesion-dependent manner

**Figure 5:**
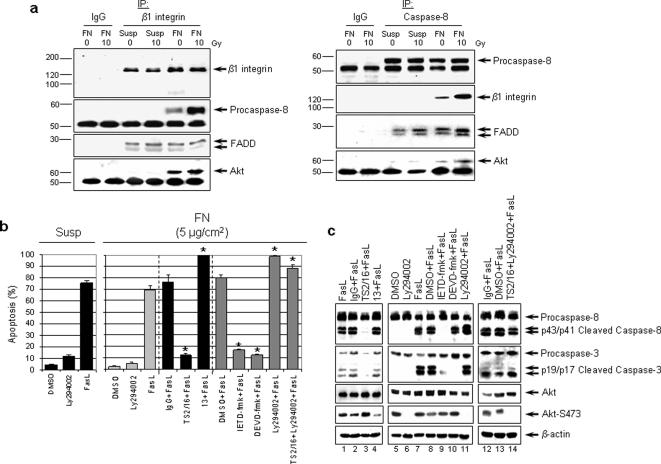
Upon adhesion, β1 integrin-mediated antiapoptotic signaling involves procaspase-8 and Akt. (a) Coprecipitation was performed to detect interactions between β1 integrin and procaspase-8, FADD or Akt. Cells were prepared as described in Materials and Methods and immunoprecipitation (reverse immunoprecipitation used anti-caspase-8 mAb) was carried out at 4 h after irradiation using non-specific IgG or anti-β1 integrin antibodies. (b) To analyze the impact of procaspase-8, -3 or Akt on the induction of apoptosis following FasL, HL60VC cells were held in suspension or plated onto FN and, where indicated, incubated with mAb TS2/16 or 13 (anti rat IgG1 as control) for 1 h. After 30 min, cells were also exposed to 20 µM of inhibitors for caspase-8 (IETD-fmk), -3 (DEVD-fmk), 10 µM Ly294002 (PI3K) or 0.25 µl/ml DMSO. After additional 30 min, treatment with 300 ng/ml FasL was accomplished and cells were isolated, stained with DAPI and counted for apoptotic morphology at 48 h thereafter (mean±s.d.; n = 3). Statistics were calculated by comparing inhibitor-treated cells versus DMSO or IgG. *P<0.01. (c). In parallel, total cell extracts were isolated, subjected to Western blotting and pro and cleaved forms of caspase-8 and -3 and Akt and Akt-S473 were detected using the appropriate antibodies. β-actin was the loading control.

Coprecipitation experiments were performed showing a similar amount of precipitated β1 integrin or FADD under suspension and FN adhesion (Figure 5a). In contrast, procaspase-8 and Akt were increasingly observable in β1 integrin precipitates in irradiated HL60VC FN cultures relative to non-irradiated controls; a finding confirmed by reverse immunoprecipitation (Figure 5a). It remains unclear whether β1 integrin directly or indirectly interacts with procaspase-8 and Akt and which role FADD plays in this scenario. Therefore, HL60β1 cells were treated with FasL.

Similar to suspension conditions (Figure 5b), FasL strongly induced apoptosis in HL60β1 cells grown on 5 µg/cm^2^ FN, a concentration ineffective to diminish apoptosis induction after irradiation or Ara-C in these cells (Figure 5b). However, stimulation of β1 integrins using TS2/16 significantly (*P*<0.01) decreased the rate of apoptosis relative to IgG and in contrast to mAb13 (Figure 5b). Inhibitors of procaspase-8 (IETD-fmk) or -3 (DEVD-fmk) prevented apoptosis in FasL-treated FN cultures. In contrast, PI3K inhibition by Ly294002 promoted FasL-mediated apoptosis that could only be insufficiently antagonized by TS2/16 acting on upstream localized β1 integrins (Figure 5b). Caspase cleavage and Akt phosphorylation under identical conditions exhibited that β1 integrin stimulation with TS2/16 abrogated procaspase-8 and -3 cleavage in parallel to increased Akt-Ser473 phosphorylation by FasL (lane 3) relative to controls (lane 1 and 2) (Figure 5c). In contrast, mAb13 inhibited phosphorylation of Akt-Ser473 while procaspase-8 and -3 were strongly cleaved (lane 4). Ly294002 abolished Akt phosphorylation (lane 6) and increased procaspase-8 cleavage in FasL-treated controls (lane 11) (Figure 5c). Inhibitors of procaspase-8 and -3 blocked cleavage of their cognate procaspases under FasL stimulation without affecting Akt (lane 9 and 10) (Figure 5c). A TS2/16-Ly294002 combination reduced cleavage of procaspase-8 while active procaspase-3 and Akt-Ser473 remained constant in FasL-treated HL60β1 FN cultures (lane 14) as compared to IgG and DMSO (lane 12 and 13) (Figure 5c).

### Procaspase-8 is critical for antiapoptotic effects of β1 integrins

**Figure 6:**
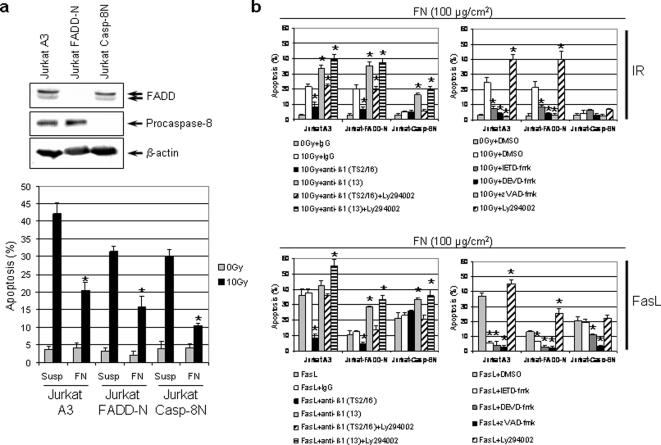
Procaspase-8 deficiency greatly decreases radiation-induced apoptosis in FN attached cells. (a) Expression of procaspase-8, FADD and β-actin was analyzed by Western blotting. Procaspase-8 negative (Casp-8N), FADD negative (FADD-N) and Jurkat A3 cells were irradiated with 10 Gy in suspension or under adhesion to 100 µg/cm^2^ FN. (b) Casp-8N, FADD-N and Jurkat A3 cells were exposed to mAb TS2/16 or mAb13 (1 µg/ml; anti rat IgG1 as control) for 1 h or 20 µM caspase-8 (IETD-fmk), caspase-3 (DEVD-fmk), pan-caspase inhibitor (zVAD-fmk) or 10 µM Ly294002 for 30 min when adhered to 100 µg/cm^2^ FN. Subsequently, cells were treated with 10 Gy or 300 ng/ml FasL. After 48 h, the number of apoptotic cells was determined by DAPI staining and counting. Columns represent mean±s.d. (n = 3). Statistical analysis was performed by comparing treatment conditions versus controls. *P<0.01.

To examine whether the β1 integrin-related antiapoptotic signals are channeled via procaspase-8 and Akt, Jurkat A3 cells deficient for procaspase-8 (Casp-8N) or FADD (FADD-N) were employed after inspection of protein expression (Figure 6a). Casp-8N cells demonstrated less radiation-induced apoptosis than FADD-N or A3 Jurkat cells (Figure 6a). All three cell lines showed significantly (*P*<0.01) diminished levels of apoptosis by FN adhesion. Similar to HL60 cells, irradiation of cells adherent on 100 µg/cm^2^ FN showed that mAb TS2/16, in contrast to mAb13, enhances the antiapoptotic action of β1 integrins relative to IgG (Figure 6b). This effect was only detectable in procaspase-8 proficient cells but not in Casp-8N cells. Without affecting Casp-8N cells, incubation of A3 and FADD-N cells with zVAD-fmk, IETD-fmk or DEVD-fmk showed a significant (*P*<0.01) decline in radiation-induced apoptosis relative to DMSO. Apoptosis in FasL-treated A3 and FADD-N FN cell cultures significantly (*P*<0.01) decreased by TS2/16, IETD-fmk, DEVD-fmk and zVAD-fmk (Figure 6b). zVAD-fmk and DEVD-fmk effectively reduced while mAb13 induced FasL-mediated apoptosis in Casp-8N cells. These data suggest that procaspase-8 is critical to radiation- and FasL-induced apoptosis under adhesion to FN in the examined cell lines.

### Knockdown of β1 integrin sensitizes cells to radiation- and FasL-induced apoptosis

**Figure 7:**
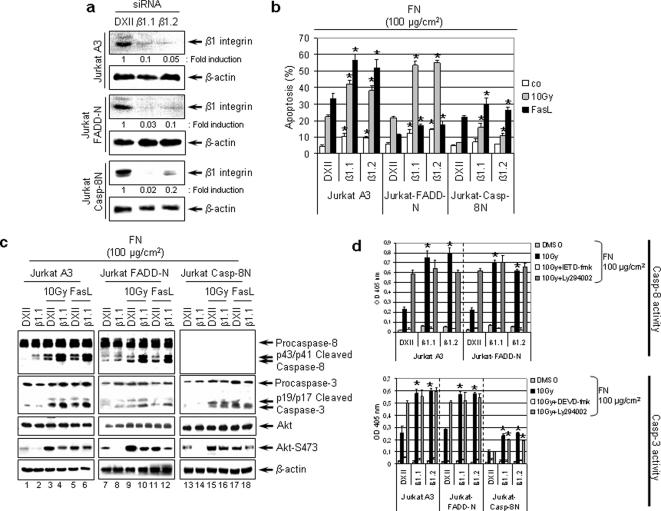
siRNA-mediated knockdown of β1 integrin sensitizes parental, FADD-N and Casp-8N cells to radiation-induced apoptosis. (a) Jurkat cell lines were transfected with two different β1 integrin (β1.1, β1.2) siRNAs or a non-specific Duplex XII (DXII) siRNA. Expression of β1 integrin was inspected by immunoblotting. (b) Following β1 integrin knockdown, 10 Gy or 300 ng/ml FasL were applied to the cells grown on 100 µg/cm^2^ FN. Apoptosis was determined 48 h later by DAPI. (c) In parallel, cell lysates were harvested for analysis of procaspase-8, -3 and Akt expression. (d) Subsequent to administration of 20 µM caspase-8 (IETD-fmk) or caspase-3 (DEVD-fmk), 10 µM Ly294002 or 0.25 µl/ml DMSO for 30 min, caspase-8 and -3 activity was measured at 4 h after 10 Gy. Statistics were calculated by comparing the level of apoptosis in β1 integrin knockdown cells versus DXII. *P<0.01.

For characterization of β1 integrin/procaspase-8/Akt interactions, we next performed siRNA-mediated knockdown of β1 integrin prior to X-ray or FasL exposure. Two different siRNAs (β1.1, β1.2) mediated β1 integrin silencing (β1.1: 90–98% repression; β1.2: 80–95% repression) relative to non-specific Duplex XII (Figure 7a). At maximum knockdown, i.e. 48 h after transfection with β1.1 or β1.2 siRNA, particularly A3 and FADD-N and to a lesser degree Casp-8N FN cultures showed significant (*P*<0.01) increase in apoptosis after 10 Gy or 300 ng/ml FasL relative to Duplex XII controls (Figure 7b). Basal levels of apoptosis merely raised in β1 knockdown A3 and FADD-N cells. Western blot analysis revealed that β1 integrin knockdown induced procaspase-8 cleavage and reduced phosphorylated Akt-Ser473 (lane 2, 8, 14) (Figure 7c). Following irradiation or FasL, β1 integrin silencing led to elevated procaspase-8 cleavage and diminished phospho-Akt-Ser473 (lane 4, 6, 10, 12) (Figure 7c). While only treated FADD-N cells demonstrated raised procaspase-3 cleavage after β1 integrin knockdown (lane 10, 12) relative to controls (lane 9, 11), procaspase-3 processing remained unaffected in irradiated or FasL-exposed A3 and Casp-8N cells (lane 4, 6, 16, 18) (Figure 7c). These data suggest a dependency of procaspase-8 activation on β1 integrin, which seems critical for initiation of apoptosis by radiation or FasL. Moreover, it indicates an inverted relationship between cleavage of procaspase-8 and Akt-Ser473 phosphorylation.

### Signaling of β1 integrin via PI3K/Akt regulates caspase-8 or -3 activation

In contrast to Duplex XII controls, caspase-8 activity in FN adherent and irradiated A3 or FADD-N Jurkat cells was significantly (*P*<0.01) increased upon β1 integrin knockdown (Figure 7d). Incubation of cells with IETD-fmk or DEVD-fmk prior to irradiation effectively blocked caspase-8 or -3 activation, respectively. Ly294002, however, abrogated the reduced caspase activity both in irradiated Duplex XII controls as well as β1 integrin knockdown cells grown on FN Figure 7d). Casp-8N cells showed less caspase-3 activation than FADD-N and A3 cells.

## Discussion

Chemo- and radiotherapeutic responses of leukemia cells are essentially modified by integrin-mediated adhesion to extracellular matrix [5], [39]. In general, integrin-mediated resistance to cytotoxic stimuli is well-known but the underlying molecular mechanisms still remain unsolved. Our findings show in detail that leukemia cells adherent to fibronectin, laminin or collagen-1, which represent β1 integrin ligands, are protected from radiation, Ara-C or FasL-induced apoptosis. These β1 integrin-mediated cell-matrix interactions inhibit procaspase-8 activation via complex formation with Akt in a PI3K dependent manner. Additionally, β1 integrin ligation to FN provides stabilization of the mitochondrial transmembrane potential and impairs both procaspase-3 and -9 activation associated with the intrinsic apoptotic pathway.

Unexpectedly in its extent, increases in β1 integrin total and cell surface expression inevitably required increased availability of a ligand, here fibronectin, laminin or collagen-1, for sufficient antiapoptotic action after different types of cell stress such as ionizing radiation, FasL or Ara-C. To note, serum depletion also reduced the rate of radiation-induced apoptosis. It can be hypothesized that specific growth factors are critical for the accurate execution of proapoptotic pathways. Extensive experiments have already commenced in our laboratory to elucidate this observation in more depth. By parallel modulation of both the intrinsic and extrinsic apoptotic pathway, the functional duality of the integrin β1 subunit in prosurvival processes is exceptionally displayed in our study.

Owing to recent findings on procaspase-8 in integrin-mediated death [22] and cell adhesion-mediated drug resistance [24], we explored a possible interplay between β1 integrin and procaspase-8. The use of stably transfected β1 integrin-overexpressing HL60 cells attached to increasing concentrations of fibronectin enabled us to observe a new role for procaspase-8 in radiation-induced apoptosis. Moreover, it became clear that the antiapoptotic effect mediated by β1 integrins is tightly associated with the amount of ligand bound to β1 integrins expressed on the cell surface. Thus, unligated β1 integrins signal via yet unknown pathways for induction of apoptosis, which might be an effective mechanism for cell removal under specific pathological or physiological circumstances. Although this cellular phenomenon has already been described in adherent growing cells and termed integrin-mediated death [22], our data point out the similarity between adhesion and suspension cell cultures with respect to the cellular susceptibility to integrin signals. Most interestingly, the β1 integrin/procaspase-8/Akt complex showed to be crucial for cell survival after different stressors such as ionizing radiation, Ara-C or FasL. Exclusively under FN adhesion, procaspase-8 was increasingly detectable in the β1 integrin coprecipitate in irradiated cells. Akt, colocalized in this complex, showed a similar pattern. The data suggest that a β1 integrin/procaspase-8/Akt interrelation already exists when cells are adherent to FN. After cytotoxic stress, this interaction seems to be propagated, which is shown by a higher content of β1 integrin, procaspase-8 and Akt in the coprecipitate. Further experiments exposing cells to more specific inhibitors for procaspase-8, -3 and PI3K and anti-β1 integrin stimulatory or inhibitory mAbs underlined this hypothesis.

To evaluate these effects in Jurkat cells deficient for the critical molecules of the death receptor cascade, i.e. procaspase-8 and FADD, procaspase-8 and FADD deficient cells were tested. FADD-negative cells reacted, in general, similar to A3 Jurkat control cells under adhesion conditions. Blocking caspase activation by pharmacological inhibitors reduced radiation- and FasL-induced apoptosis in contrast to Ly294002. PI3K deactivation resulted in elevated levels of apoptosis under all tested treatment regimes. As this indicates that the effect is procaspase-8- but not FADD-dependent, procaspase-8 deficient Jurkat cells showed less apoptosis throughout the diverse treatment and growth conditions tested but retained some of their susceptibility to β1 integrin modification by anti-β1 mAbs. Despite data that describe FADD recruitment to FasR in the absence of FasL for activating procaspase-8 after anticancer drugs or UV-irradiation [40]–[42], our observations do not indicate FADD to be critical for the regulation of radiation-induced apoptosis in FN adherent Jurkat cells. Our observations strongly argue for an Akt-dependent antagonization of procaspase-8 that is independent from FADD.

Accomplishing knockdown of β1 integrin by siRNA increased the sensitivity of Jurkat A3, FADD-N, and Casp-8N cells particularly to X-rays and to a lesser extent to FasL. In addition to pronounced augmentation in caspase-8 and -3 activity, elevated cleavage of procaspase-8 and -3 was associated in all cases with an attenuated phosphorylation of Akt at S473.

In summary, our data demonstrate, for the first time as to our knowledge, a regulatory interaction between β1 integrin, Akt and procaspase-8 selectively assembled after integrin-mediated adhesion of leukemia cells to FN. Due to its critical role in interfering with apoptosis-triggering agents such as ionizing radiation, FasL and Ara-C, this complex might essentially contribute to pre-existing or acquired resistance mechanisms effectively counteracting current antitumor therapies. Both, agents targeting β1 integrin signaling and agents targeting the PI3K/Akt pathway might represent potent novel adjuvant therapeutic options. Application of such agents in conjunction with conventional therapies might effectively reduce drug-resistant tumor populations and treatment failure in hematological malignancies.

## Materials and Methods

### Reagents, antibodies and cell culture

All reagents were purchased from commercial sources: 4′,6 Diamidino-2-phenylindole (DAPI; Serva, Heidelberg, Germany), FITC-VAD-fmk (Promega, Mannheim, Germany), TMRE (tetramethylrhodamine, ethyl ester, perchlorate), MitoTracker® Red CMXRos (Molecular Probes, Leiden, Netherlands), zVAD-fmk, Ly294002, protein-G-agarose beads, diaminobenzidine (Sigma, Taufkirchen, Germany), DEVD-fmk, IETD-fmk (Chemicon, Hampshire, UK), GRGDS (H-Gly-Arg-Gly-Asp-Ser-OH) and GRADSP (H-Gly-Arg-Ala-Asp-Ser-Pro-OH) peptides, G418 (Calbiochem, Bad Soden, Germany), Vectashield® medium (Alexis, Grünberg, Germany), nitrocellulose membranes (Schleicher and Schuell, Dassel, Germany), ECL (Amersham, Freiburg, Germany). Antibodies used are: anti-β1 integrin (TS2/16; Perbio, Bonn, Germany), anti-rat IgG1, anti-mouse IgG1 (Upstate, Hamburg, Germany), anti-caspase-3 cleaved, anti-caspase-3, anti-caspase-9 cleaved, anti-caspase-9, anti-caspase-8 cleaved, anti-caspase-8, anti-PARP cleaved, anti-PARP, anti-FADD, anti-Akt-S473, anti-Akt (Cell Signaling, Frankfurt a.M., Germany), anti-β-actin (Sigma, Taufkirchen, Germany), anti-β1 integrin (BD, Heidelberg, Germany); HRP-conjugated goat anti-rabbit and anti-mouse antibodies (Santa Cruz, Heidelberg, Germany). Anti-β1 integrin (13) was a generous gift from K.M. Yamada (Bethesda, MA, USA). FITC conjugated anti-β1-integrin IgG and FITC-conjugated non-specific anti-IgG antibodies were from Dako (Hamburg, Germany). Anti-Histon H3 was from Acris (Hiddenreich, Germany). Human promyelocytic HL60 leukemia and Jurkat A3 T-lymphoma cells were purchased from ATCC (Bethesda, MD, USA). Caspase-8- and FADD-deficient Jurkat A3 cells were a kind gift from P. Juo and J. Blenis (Boston, MA, USA). RPMI-1040 GlutaMAX 1TM supplemented with 1% non-essential amino acids (GIBCO, Karlsruhe, Germany) and 10% FCS (PAA, Linz, Austria) was applied to culture the cells routinely at 37°C-5% CO2, pH 7.4. Serum starvation of cells was performed using RPMI-1040/1% non-essential amino acids without FCS. For all experiments, asynchronous growing cell cultures were used.

### Construction of mammalian β1 integrin expression vector and DNA transfection

The full-length of human β1 integrin cDNA was generated by PCR and cloned into the pcDNA3 expression vector using EcoR1 sites (Invitrogen, Karlsruhe, Germany). Subsequent to electroporation [21], selection was performed under 1000 µg/ml G418. The expression of β1 integrin in transfectants was confirmed by Western blotting. Stable transfectants were pooled and used as a population designated HL60β1 and HL60VC. All constructs were sequence verified at IMGM Laboratories GmbH (Martinsried, Germany).

### Cytotoxicity assays

Cells were induced to undergo apoptosis using ionizing radiation, Ara-C or FasL. Cells were grown in suspension (polystyrene, BSA (bovine serum albumin)) or on FN (BD, Heidelberg, Germany) plus/minus serum for 1 h, irradiated or left unirradiated, treated with Ara-C (0, 5 nM, 5µM) or FasL (300 ng/ml; Merck, Germany), prepared by cytospin, washed with 0.9% NaCl and permeabilized using 4% paraformaldehyde for morphological evaluation of apoptosis by DAPI staining as published [21]. Irradiation was delivered at room temperature using single doses of 240 kV X-rays (Isovolt 320/10; Seifert, Ahrensburg, Germany) filtered with 3 mm Beryllium. The absorbed dose was measured using a Duplex dosimeter (PTW, Freiburg, Germany). The dose-rate was approximately 1 Gy/min at 13 mA. FN attached cells were removed using Trypsin/EDTA solution (GIBCO, Karlsruhe, Germany). Following DAPI staining, 10^3^ cells were enumerated using a Leitz Diaplan microscope (Bensheim, Germany). Cells were counted by three independent observers (D.E., A.F., N.C.). Interobserver variation was<5%. Where indicated, cells were incubated with 20 µM pan-caspase (zVAD-fmk), caspase-3 (DEVD-fmk), -8 (IETD-fmk) or 10 µM PI3K (Ly294002) inhibitor or 0.25 µl/ml DMSO vehicle for 30 min prior to antibody or peptide exposure and/or treatment. TS2/16, 13, IgG, GRGDS or GRADSP incubation was accomplished in parallel to seeding cells on polystyrene or FN prior to treatment. Further, cell viability was quantified using a 3-(4,5-dimethylthiazol-2-yl)-2,5-diphenyltetrazolium bromide (MTT) colorimetric assay. Briefly, cells were seeded in uncoated or FN-precoated 96-well plates (3 x 10^4^/mL) overnight and irradiated or treated with Ara-C for 48 h. MTT (Roche, Mannheim, Germany) solution was added to each well and incubated for 4 hours at 37°C. The supernatant was removed, and the MTT-formazan crystals formed by metabolically viable cells were dissolved in Solubilization solution. Absorbance at 550 nm and 690 nm was monitored using a spectral-photometer (Spectra max^®^ 190, Molecular devices, Ismaningen, Germany).

### Limiting dilution analysis

In average, one cell was plated in every non-coated or FN- or BSA-precoated well of a microtiter plate. After 1 h, irradiation (0, 2, 4, 6 Gy) or Ara-C (48 h; 0, 5 nM, 5 µM) was delivered and cells were allowed to grow for 8 days according to Grenman et al. [43]. Proliferation of cells was determined by microscopy and scored for significant cell growth defined as positive wells. Positive wells were counted and surviving fractions were calculated in relation to non-irradiated or non-Ara-C-treated controls.

### Protein fractionation

For fractionation of membrane, cytoplasmic and nuclear proteins, cells were lysed in lysis buffer (50 mM Tris-HCl (pH 7.5), 10 mM MgCl_2_, 5 mM EDTA, protease inhibitor cocktail complete® (Roche, Mannheim, Germany)) and sonicated (2×1 sec, level 4, 60%) on ice and cytoplasmic proteins were separated from nuclear and membrane proteins by centrifugation (100,000×g, 15 min, 4°C). Then, the pellet was resuspended in Triton X-100 buffer (1% Triton X-100, 10 mM MgCl_2_, 0.2 mM Na_3_VO_4_, protease inhibitor cocktail complete®) to separate membrane proteins from nuclear proteins by centrifugation (23,000×g, 5 min, 4°C). After removal of the supernatant containing the membrane faction, the pellet was resuspended in loading buffer (50 mM Tris-Base (pH 6.8), 2 ml Glycerol, 10% SDS, 0.5 ml β-mercaptoethanol, 1 mg bromphenol blue). Each protein fraction separated by Western blotting contained the protein amount from 2×10^5^ cells. To verify accurate protein fractionation, Histon H3 was detected in the nuclear fraction and a lactate dehydrogenase (LDH) assay (Roche, Mannheim, Germany) was performed on the cytoplasmic fraction. Samples were prepared according to the manufacturer's instructions. Absorbance at 490 nm and 690 nm was monitored using a spectral-photometer (Spectra max^®^ 190).

### Integrin analysis by flow cytometry

The expression level of transfected β1 integrins was measured by FACS analysis as published [26]. Staining with FITC-conjugated β1 integrin IgGs or FITC-conjugated, isotype-matched non-specific control IgGs was achieved for 1 h at room temperature. Finally, prepared cells were resuspended and the FL1 (green fluorescence) was measured from 10^4^ events using a fluorescence-activated cell sorter (FACS) Calibur (BD, Heidelberg, Germany) equipped with a CELLQuest software (BD, Heidelberg, Germany).

### Adhesion assay

Cell adhesion to FN was studied according to a previously published method [44].

### Measurement of the ΔΨm

At 24 h after treatment, cells were prepared for measurement of the ΔΨm using 25 nM TMRE and flow cytometry following the manufacturer's instructions as published [21]. Subsequent to a 30-min staining and washing, cells were resuspended and acquisition and analysis of data for 10^4^ events was performed using a FACS Calibur. The radiation-, Ara-C and substratum-dependent changes of the MTP were analysed from dot plots and histograms after exclusion of necrotic cells based on forward and side scatter criteria using CELLQuest software.

### Detection of activated caspases

Analysis of activated caspases was performed as previously described using FITC-VAD-fmk and flow cytometry [21]. At indicated time points, cells were centrifuged, washed with phosphate-buffered saline and incubated with FITC-VAD-fmk for 20 min. After washing, cells were resuspended and acquisition of data for 10^4^ events was performed after exclusion of necrotic cells based on forward and side scatter criteria using a FACS Calibur and CELLQuest software.

### Total protein extraction and Western blotting

After 10-Gy X-rays or 300 ng/ml FasL, suspension and FN adhesion cultures were harvested and lysed on ice using 50 mM Tris-HCL (pH 7.4), 1% NP-40, 0.25% sodium deoxycholate, 150 mM NaCl, 1 mM EDTA, protease inhibitor cocktail complete®, 5 mM sodium vanadate and 5 mM sodium fluoride. Amounts of total protein extracts were determined using BCA assay (Interchim, Montlucon Cedex, France) and samples were stored at −134°C until use. Western blotting was performed as described previously [26]. Measurements of protein band density were carried out using ImageQuant version 5.0 software (Molecular Dynamics, Germany).

### Caspase-3 and -8 activity assay

Caspase-3 or -8 activities were measured after 10-Gy X-rays plus/minus DEVD-fmk or Ly294002 (control: DMSO) in triplicates using a commercially available ApoAlert assay kit (BD-CloneTech, Heidelberg, Germany) or Caspase-8 Colorimetric Activity Assay Kit (Chemicon, Ochsenhausen, Germany) according to the manufacturer's instructions. Experiments were repeated three times.

### Coprecipitation experiments

Cells were grown in suspension or on 100 µg/cm^2^ FN in serum-free medium 1 h before 10-Gy radiation. Then, cells were treated for 15 min with 1% formaldehyde to crosslink proteins, a reaction terminated with 100 mM glycine. Following cell lysis, β1 integrin was immunoprecipitated with 2 µg of the specific antibody overnight at 4°C from 250 µg total protein extracts. Subsequently, protein-G-agarose beads were allowed to incubate for 3 h, followed by washing and preparation for SDS-PAGE. β1 integrin and coprecipitated procaspase-8, FADD or Akt were detected by Western blotting. Non-specific mouse-IgG was used as control.

### siRNA transfection

The target sequences that effectively mediate silencing of β1 integrin expression are 5′-AATGTAACCAACCGTAGCA-3′ (β1.1) and 5′-GCGCATATCTGGAAATTTG-3′ (β1.2) (sense sequences) as reported previously [44]. The 21-nucleotide synthetic siRNA duplex was prepared by MWG (Ebersberg, Germany) based on Dharmacon 2′-ACE technology. Jurkat cells were transfected with the β1 integrin siRNA or a 21-nucleotide irrelevant RNA Duplex XII as a control using oligofectamine (Invitrogen, Karlsruhe, Germany). Depletion of β1 integrin was confirmed by Western blotting.

### Data analysis

Means±s.d. of three independent experiments were calculated with reference to untreated controls defined in a percentage scale or 1.0. To test statistical significance, Student́s *t* test was performed using Microsoft®Excel 2000. Results were considered statistically significant if *P*-value of less than 0.05 was reached. All experiments were repeated at least three times.
